# The Skeletal Stability of Combined Surgery First Approach and Clear Aligners in Skeletal Class III Malocclusion Correction: A Randomized Controlled Trial

**DOI:** 10.3390/jcm13030872

**Published:** 2024-02-02

**Authors:** Meng Li, Shunyao Shen, Jingyang Huang, Yiming Wang, Jiahao Bao, Bo Wang, Hongbo Yu

**Affiliations:** 1 Department of Oral and Craniomaxillofacial Surgery, Shanghai Ninth People’s Hospital, College of Stomatology, Shanghai Jiao Tong University School of Medicine, Shanghai 200025, China; 2 National Center for Stomatology, National Clinical Research Center for Oral Diseases, Shanghai 200011, China; 3 Shanghai Key Laboratory of Stomatology, Shanghai Research Institute of Stomatology, Shanghai 200011, China

**Keywords:** surgery first approach, clear aligners, skeletal class III malocclusion, skeletal stability, treatment duration

## Abstract

The surgery first approach (SFA) and clear aligners technique can address traditional treatment defects, such as prolonged waiting times for surgery and a less desirable facial appearance due to wire aligners. However, the curative effect of the combination remains uncertain. The randomized controlled study aimed to evaluate the skeletal stability of the SFA compared to the conventional orthodontic first approach (OFA), both of which were applied with clear aligners. A total of 74 participants were randomly allocated to two groups: the SFA group (experimental) and the OFA group (control). The skeletal deviation was calculated using reconstruction models from computed tomography scans taken immediately and 6 months after surgery. The largest median deviations were detected in the y-axis of the mandible for both two groups, separately 1.36 mm in the experimental group and 1.19 mm in the control group. Apart from the maxillary yaw dimension (*p* = 0.005), there were no significant differences between the two groups in terms of linear and angular deviation. The experimental group had an overall treatment time of 18.05 ± 2.53 months, while the control group took 22.83 ± 3.60 months (*p* < 0.05). Therefore, the combined surgery-first and clear aligners treatment can achieve comparable skeletal stability to the conventional approach, while also saving significant time.

## 1. Introduction

Skeletal class III malocclusion holds a higher incidence in Asia than in the Caucasian population according to the previous publication, especially in Japan and Korea [1], and these patients represent maxillary discrepancy and mandibular excess, which imbalanced facial appropriation and destroyed natural appearance. The minority can be treated with orthodontic camouflage for compromised skeletal position and feasible occlusion. For the majority of patients with severe malocclusions and apparent facial deformities, combined surgery and orthodontic treatment should be performed to anatomically correct malposition and further stabilize the interdental relationship.

The conventional integrated approach included presurgical orthodontics, orthognathic surgery (OGS), and postsurgical orthodontics, and each part of the project is conducted sequentially according to a plan formulated by an orthodontist and surgeon team. All these procedures take a lot of time, especially the pre-surgical orthodontic part, which leaves the patient with a lower quality of life and an even worse appearance due to the wires. On the other hand, the long waiting time before surgery cannot satisfy the patients’ eagerness to change their facial appearance, and some of them are discouraged from continued treatment. Consequently, the avoidance of presurgical orthodontics has been attempted. Surgery first approach (SFA) was proposed in the last century and has been well implemented throughout the world to treat skeletal malocclusion [2,3,4,5]. With no presurgical orthodontics, orthognathic surgery is performed to correct the skeletal discrepancy, and postsurgical orthodontics proceeds to manipulate alignment, which is a feasible method on both theoretical and practical grounds [6,7]. It has distinct advantages, shich as no need for presurgical orthodontics, which deeply saves time and can realize obvious changes in appearance in a short period.

Except for treatment duration, another factor that both doctors and patients worry about is the discomfort and aesthetic impact of orthodontic procedures. Recently, in response to the demand for aesthetics, clear aligners have become popular. It has shown that clear aligners were superior in terms of treatment outcomes, especially in cases where molars were pushed back, and were more aesthetically pleasing than arch wires [8,9]. Therefore, the combination of clear aligners and surgery first approach can solve both time-consuming and aesthetic problems in combined orthognathic orthodontic treatment, which deserves to be applied in clinics.

However, whether the postsurgical skeletal stability of SFA differs from conventional treatment, or not, is still ambiguous, which is a significant footstone that identifies that SFA can realize the same effects as the conventional approach. Previous studies that focused on comparing the stability of the two approaches were mostly retrospective and did not obtain definitive conclusions [10]. Above all, this study aimed to combine invisible orthodontics with the surgical first approach for the treatment of skeletal Class III malocclusion and to identify the skeletal stability deviation between this concept and the orthodontics-first approach through a prospective randomized controlled method.

## 2. Materials and Methods

### 2.1. Study Design and Sample

To achieve the study objectives, a prospective, randomized, controlled, and nonblinded trial was designed. The ethics approval was obtained by the hospital institutional board of Shanghai Ninth People’s Hospital, Shanghai Jiao Tong University (SH9H-2019-T129-2). All the participants signed the informed consent agreement. The prospective randomized controlled trial was registered at the Chinese Clinical Trail Registry website (Registration number: ChiCTR2000033553).

From March 2020 to January 2023, the patients were selected according to inclusion and exclusion criteria in the Department of Oral and Maxillofacial Surgery, Shanghai Ninth People’s Hospital, China. All eligible participants were randomly assigned into two groups (surgery first group and orthodontic first group) with a ratio of 1:1. The randomization process was conducted strictly according to a randomization table generated by a Microsoft Office Excel 2013 spreadsheet (Redmond, Wash.). A particular member kept the randomization table and followed the implementation when a new participant was recruited, and then he or she was allocated to the randomized group. The member did not anticipate the treatment operation. The inclusion criteria were: (1) 18 years or older; (2) diagnosed as skeletal Class III malocclusion with mild chin deviation (<3 mm); (3) available dental conditions for performing clear aligners and the patient’s consent on the application of Invisalign; (4) the patient’s functional and facial deformity can be addressed by both surgery first approach and conventional orthodontic first treatment; and (5) indication of two-jaw operations including Le Fort I and bilateral sagittal split ramus osteotomies. The exclusion criteria were: (1) craniofacial syndromes; (2) random allocation harms the treatment outcomes; and (3) patients on periodontitis active stage.

The sample size was calculated by PASS 19 software (NCSS Statistical Software, Kaysville, UT, USA). According to previous research, the postoperative skeletal deviation was 1.6 ± 2.4 mm in the surgery first group and 1.6 ± 2.3 mm in the orthodontic first group [4]. Based on a two-tailed alpha value of 0.05, a power of 0.8, and a dropout rate of 10%, the sample size was 41 cases for each group. Eventually, a total of 74 participants completed follow-up and data analysis, including 37 cases in each of the two groups. The demographics were shown in Table 1 and comparability between the two groups was ensured.

### 2.2. Interventions

**Figure 1:**
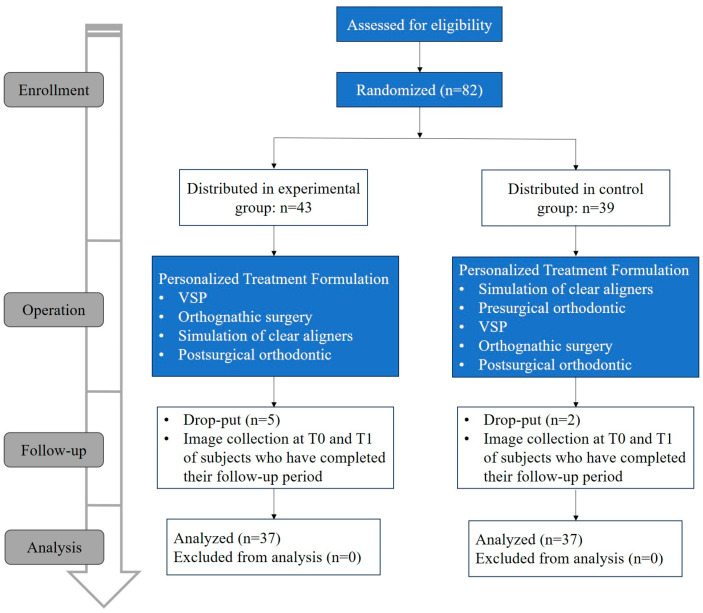
Simplified diagram of the randomized controlled trial implementation process. T0, immediate after surgery; T1, 6 months after surgery.

The participants eligible for our study were randomly distributed to two groups, the surgery first group (SFA group, experimental group) and the orthodontic first group (OFA group, control group). The SFA group received the intervention, i.e., the surgical first treatment. The OFA group did not take any intervention and routinely followed the orthodontic preferred treatment model. For both groups of patients, the computed tomography (CT) scans and dental cast models were acquired before intervention to evaluate their skeletal and occlusal condition for further operation. The CT was taken by a voxel size of 0.45 mm × 0.45 mm, a slice interval of 1.25 mm, and a resolution of 512 × 512 × 231 (LightSpeed Ultra 16 spiral CT machine, GE Company, Boston, MA, USA). After primary estimation by the experts of our team, the treatment schedule of participants of two groups were formulated, respectively (Figure 1).

#### 2.2.1. Experimental Group

The participants in the experimental group leaped over the presurgical orthodontic section and proceeded directly to orthognathic surgery following the completion of preoperative preparations. Prior to surgery, CT scans and digital dental scans were taken into ProPlan CMF 3.0 software (Materialise, Leuven, Belgium) for virtual surgery planning (VSP). The final occlusion and orthognathic procedure were confirmed by digital simulation, upon which the manufactured splints were based. During surgery, the maxilla was firstly separated along the Le Fort I osteotomy line, and the position of the inferior maxilla was refixed through the splints, using titanium plates and cortical bone screws. Then, bilateral sagittal split ramus osteotomies were carried out, and the segmented mandible was repositioned along final splints with plates and screws. The final splints have remained in maxilla to maintain the present occlusion for a month, aided by intermaxillary elastic distraction. The splints were removed one month after surgery, and subsequently, the postoperative orthodontic treatment was activated. The intra-oral scans, taken by iTero Element intraoral scan (Align Technologies, San Jose, CA, USA), were processed to obtain new occlusion to develop clear aligners in ClinCheck Pro 5.0 software (Align Technology, Santa Clara, CA, USA). After digital simulation and personalized orthodontic design, the clear aligners manufactured by the factory were dispatched to the patients, who were instructed to wear aligners at least 20 h per day and changed to the next pair sequentially every 10 days. The orthodontist needed periodic appointments of patients every two months until all of the clear aligners were applied.

#### 2.2.2. Control Group

All participants assigned to the control group formulated a treatment plan with ordered preoperative orthodontic, OGS, and postoperative orthodontics. So, following the schedule development step, the orthodontic section was started before orthognathic surgery, which differed from the experimental group. As same as SFA’s postoperative orthodontic simulation process, the digital dental models were imported into the ClinCheck software to develop a personalized orthodontic schedule. For skeletal Class III malocclusion, preoperative orthodontics principally aims to decompensate anterior teeth, coordinate dental arch width, level the curve of Spee, and close the space of extraction. The well-organized procedures were planned through the virtual orthodontic simulation and then applied by a set of clear aligners to meet the occlusal requirements for orthognathic surgery. Once the analogue alignment was achieved, the participants could proceed to the next step. Upon completing the virtual surgery simulation preparations and manufacturing of splints, participants underwent Le Fort I and bilateral sagittal split ramus osteotomy. Jaw segments were repositioned using titanium plates and cortical bone screws, much akin to the experimental group. The splints were removed one month after surgery, same as the experimental group. Before activating postoperative orthodontics, the orthodontist must reappraise the temporal occlusion to determine whether a redesign of the orthodontic process is necessary. If the alignment deviates only slightly from the previous simulation, the postoperative orthodontic treatment can proceed with the original design [11]. Otherwise, the participants must rescan the oral models and design clear aligners after the operation. The primary goal of this stage is to enhance the coordination of dental arch width and further align the dentition to preserve postoperative occlusal stability. All requirements for wearing clear aligners and follow-up intervals were identical to those of the experimental group.

### 2.3. Stability Evaluation

The primary indication of experimental and control group outcome contrast was skeletal stability deviation between the immediate moment and 6 months after surgery by reconstructed model. The secondary indication was the treatment time-consuming differentiation between the two groups. The treatment durations for the two groups were displayed in Table 1.

**Figure 2:**
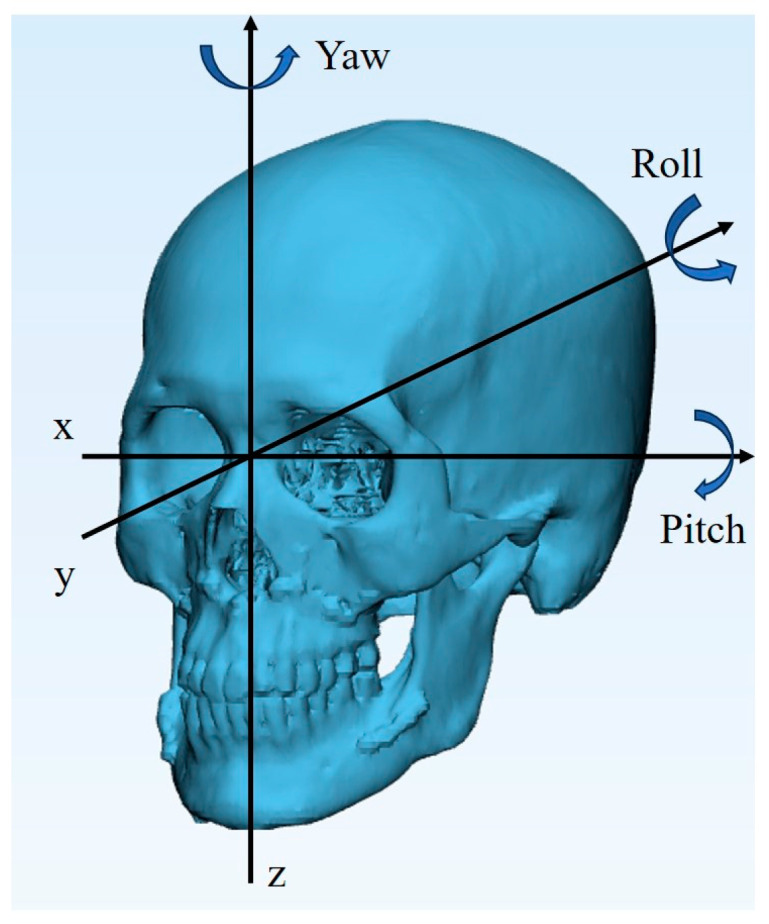
Definition of coordinate system and rotating regulations.

The CT scans taken immediately and 6 months after surgery for both groups were collected and reconstructed by ProPlan CMF 3.0 software. Two digital models were registered by the surface best-fit method [12,13]. The stable midface was set as a benchmark for aligning the maxilla since it was not repositioned during the entire treatment period. The condyle head position is relatively dependable to the superimposition of two digital mandible models. Once the two digital skulls were aligned in the same space, the spatial coordinate system was constructed based on the original CT images, to further investigate the deviation in spatial relationships that reveal postoperative skeletal stability. The mediolateral dimension, anteroposterior dimension, and superoinferior dimension were defined as the x, y, and z axes, respectively (Figure 2). After superimposing the two models, the skeletal discrepancy was reflected by a specific distance between the Le Fort I segment and the distal mandible segment of the two models, because those parts were shifted during the surgery. Subsequently, three landmarks were labelled on each segment to represent their location in the spatial coordinate system [13]. All the above operations, including superimposition and landmarks determination were, respectively, processed in the “Segment” module and the “CMF/Simulation” module of ProPlan software.

**Figure 3:**
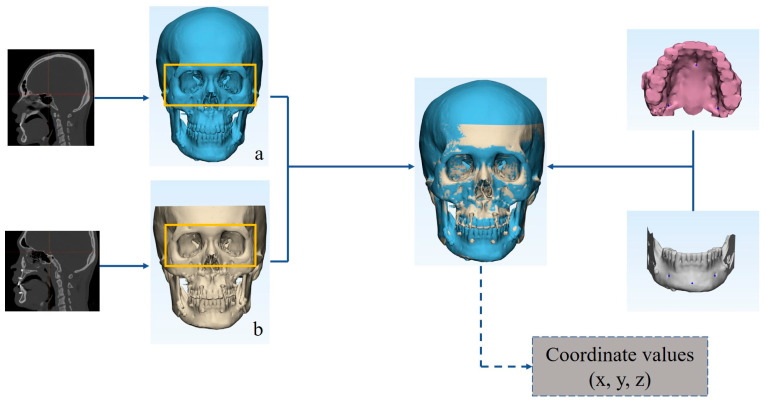
Digital workflow of the model registration and deviation arrangement originated from two sets of coordinates. (**a**) Reconstructed model of 6 months postoperatively; and (**b**) Reconstructed model of immediate postoperatively.

The maxillary landmarks were identified as the bilateral greater palatine foramen and incisive foramen. The mandibular landmarks chosen were the bilateral mental foramen and pogonion. The first step in evaluating stability involved applying these landmarks to the dissociative model, which was obtained by cutting the skull model using Le Fort I and bilateral sagittal split ramus osteotomy lines. Two segments were registered using the surface best-fit method with two CT reconstructed models. The coordinates of six landmarks were recorded for both models, allowing for two sets of coordinates to represent the position of the maxilla and mandible immediately after surgery, and six months post-surgery (Figure 3). The coordinates were calculated in MATLAB R2012b (The MathWorks Inc., Natick, MA, USA) to determine the postsurgical linear and angular deviations that represented the skeletal stability for both the experimental and control groups. The linear deviations were the orthogonal outcomes along the x, y, and z axes. The angular deviations included the roll, yaw, and pitch rotations, respectively defined as rotating along the y, z, and x axes (Figure 2).

### 2.4. Statistical Analysis

Data were imported into IBM SPSS Statistics version 25 (IBM Corp., Armonk, NY, USA), and descriptive statistics were calculated for linear and angular deviation for both two groups. Then distribution normality was tested with Kolmogorove–Smirnov and Shapiroe–Wilk tests. If the values were normally distributed, the independent sample *t*-test was used to calculate the difference between groups. If not, Wilcoxon signed-rank test was performed. A *p*-value less than 0.05 indicated a statistically significant difference.

## 3. Results

All of the 74 participants, completed surgery and orthodontic treatment successfully, and no complications or adverse events were found during the treatment and follow-up period. The surgical procedure went smoothly and took less than three hours in both two groups. The age distribution is 24.62 ± 3.89 years for the experimental group and 25.73 ± 4.64 years for the control group. The difference in the gender and age factors between the two groups was not statistically significant (Table 1), indicating that the subject characteristics were consistent.

**Figure 4:**
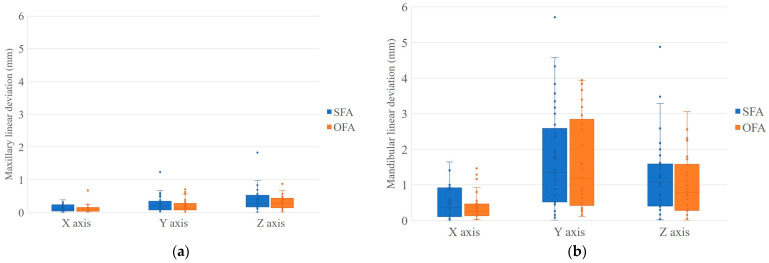

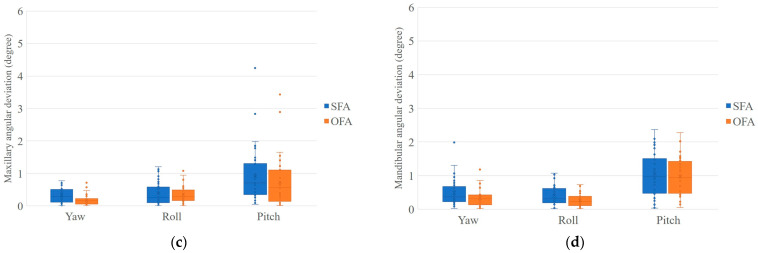
The box plots of linear and angular deviation of SFA group and OFA group. (**a**) Maxillary linear deviation; (**b**) mandibular linear deviation; (**c**) maxillary angular deviation; and (**d**) mandibular angular deviation.

The statistical description and comparative test results of the postoperative stability of the experimental and control groups are shown in Table 2 and Table 3, and Figure 4. In terms of primary outcome indication, statistical tests revealed no significant difference between the two groups in terms of linear and angular differences, except for the maxillary yaw dimension (*p* = 0.005). The linear differences in all dimensions were under 1 mm and the angular differences were under 1 degree, except in the y-axis and z-axis of the mandible, where the linear differences were above 1 mm. In the mandibular y-axis, the interquartile range was largest for both the experimental and control groups, being in the range of 0.52–2.59 mm and 0.42–2.85 mm, respectively. Referring to the secondary outcome indicators, the entire treatment time of the experimental group was 18.05 ± 2.53 months, while the control group was 22.83 ± 3.60 months. Statistical analysis demonstrated that there was a significant difference in treatment duration between the two groups (Table 1). Therefore, the surgery first approach exhibited superiority over the orthodontic first approach in shortening the treatment time.

## 4. Discussion

Skeletal malocclusion deformities manifest themselves primarily as impaired occlusion and skeletal malposition that severely compromise occlusal function and aesthetic appearance. In the very beginning, the surgeons were compelled to operate first for treating dentomaxillofacial deformities, since there was no orthodontic support at the time. Then, with the rise of aligner technology, it became common for pre-surgical orthodontic treatment to be used to remove dental obstructions prior to orthognathic surgery, allowing the surgeon to osteotomize to restore a normal facial appearance based on the preferred alignment [14]. This treatment modality has been developed into the widely used combined orthognathic and orthodontic treatment approach of today. This integrated process is distinct and it can solve many maxillofacial deficiencies that cannot be addressed by simple orthodontic or surgical procedures alone. However, to cater to the evolving demands for aesthetics in modern times, the combined treatment approach must make changes. Traditional preoperative orthodontics is a time-consuming process that fails to meet the needs of patients with dental and maxillofacial deformities who are eager to alter their facial shape [15]. To solve this issue, the surgery-first approach has been proposed. In this approach, orthodontics is performed after surgery to further address the misalignments. Patients who seek early correction prefer this approach. However, the selection criteria for the surgery first approach must be carefully considered, as it may not be as inclusive as the conventional method.

The foremost dento-maxillofacial deformity in Asia is skeletal Class III malocclusion, and surgical intervention can have a significant impact on physical appearance. This is also the reason why the surgery-first approach can be widely implemented in the Asian region. In comparison to the orthodontic first approach, it offers obvious advantages in shortening treatment duration, as the preoperative orthodontic must be performed cautiously and typically takes a considerable amount of time [16]. The primary aim during the preoperative phase of the conventional approach is to decompensate the lower incisors to achieve increased overjet for enhanced surgical mobility. Preoperative orthodontics is a very uncomfortable process and can aggravate the jaw profile, which perplexes both patients and doctors. The surgical first approach can perfectly solve the above shortcomings and perform exceptionally in other aspects [10]. In the short time of post-op, rapid acceleratory phenomenon (RAP) was observed and confirmed by the previous study [17]. It is mainly due to the temporary rearrangements of the local bone microenvironment and the balance between osteogenesis and osteolysis after osteotomy, which accelerates tooth movement during postsurgical orthodontic treatment [18]. Hence, the post-op orthodontic of surgery first approach is more effective than the pre-op procedures of the orthodontic approach.

Although surgery first has many bright spots, it is not a suitable option for every patient. Due to the lack of preoperative orthodontic treatment, the dental arches of patients undergoing surgical first treatment are subject to relatively strict requirements. In general, it has been observed that mild crowding, adequate overjet, and coordinated arch width are required, which means that the patient has a relatively well aligned arch and can achieve a stable postoperative occlusion [19]. In these cases, it is not necessary to perform segmented osteotomies. Otherwise, segmented osteotomy and/or extraction is obligatory. In the case of the surgery first approach, there are no dental redressals before orthognathic surgery, so it is understandable that the postoperative stable occlusion has to make a compromise within the bounds of comfort. It is of utmost importance that the final occlusion influences both the rehabilitation of occlusal function and postoperative skeletal stability. By literature review, the comparison of surgery first approach and orthodontic first approach postoperative stability is widely discussed [3,20,21], but no consensus has been reached. Insawak et al. demonstrated in a retrospective cohort study that there was no difference in mandibular stability between the surgery first and the orthodontic first approach [2]. Furthermore, the occlusal outcome was approximate in both approaches after finished postoperative orthodontic treatment. Also, there have been reports indicating that the stability of SFA is comparatively weaker than that of OFA [3].

However, our prospective randomized controlled study found that there was no significant difference in skeletal stability between the two approaches, 6 months postoperatively. Apart from the yaw dimension of the maxillary, there were no significant differences in both transverse and rotational dimensions noted between the approaches (Table 2 and Table 3). The atypical rotation of the maxilla may be attributed to a slightly discrepant posterior dental width, which was not corrected by pre-operative orthodontic treatment. This, in turn, led to a relapse of inclination in the relatively unstable molar relationship following surgery. Incorrect interdigitation habits and muscular tension can force the repositioned mandible forward, leading to relapse after surgical setback for patients with skeletal Class III malocclusion. The advance overjet should be taken into account during the planning phase of surgery to ensure that the overcompensation allows for postoperative relapse [4]. Therefore, among the deviations derived in this study, the highest values were found on the y-axis of the mandible, containing the overcorrected portion (Figure 4b). Regardless of linear and angular deviation, the values of the maxilla are generally lower than those of the mandible. In addition to the overcorrection factors mentioned above, alterations to the temporomandibular joint and the rebound of masticatory muscles after orthognathic surgery are additional contributors to decreased mandibular stability [22]. Despite the complicated factors, the relapses of postoperative skeletal correction in both the experimental and control groups did not exceed clinically acceptable limits and remained in line with previous studies [2,3,10].

Combined surgery and orthodontic treatment can effectively improve most dento-maxillofacial deformities, as indicated by previous studies demonstrating satisfied postoperative results [5,23]. However, some patients may be hesitant to undergo the treatment due to the disruptive aesthetic effects of the orthodontic process on their daily lives. Despite the availability of the surgical first approach that reduces overall treatment duration, the aesthetic damage caused by arch wire orthodontics should not be overlooked. The invisible aligner has become increasingly popular and utilized in recent years, due to its minimal aesthetic impact and advantages for periodontal maintenance [8,24,25]. Decent treatment results have been achieved using invisible orthodontic techniques, as confirmed by previous studies in both orthodontics alone and integrated treatment with surgical interventions [9,26,27,28]. However, it is uncertain whether the combination of the surgical first concept and clear aligners yields favorable treatment outcomes, as few studies have been conducted in this field [29]. To eliminate the interference of orthodontic techniques, both the experimental and control groups in this prospective study were applied with clear aligners. The outcomes showed that both groups completed the entire treatment process and achieved sufficient skeletal stability. Participants expressed a high level of satisfaction with the ease and comfort of the orthodontic procedure.

The combination of the surgical first concept and invisible orthodontics that we applied in this article holds great value as it addressed the extensive preoperative waiting time, thus reducing the overall treatment time, and improving the aesthetic damage and periodontal health disturbances associated with conventional arch wire orthodontics through the use of clear aligners. The literature review did not identify any prospective randomized controlled trials of this treatment modality. Therefore, the postoperative skeletal stability results presented in this paper are of utmost significance for subsequent studies of this treatment approach. Whereas, there were still some limitations of our current work. The current research focuses on the stability of the jaw, and the orthodontic efficiency of the teeth and the stability of the alveolar bone should be further investigated. It is generally accepted that postoperative skeletal stability distinguishes between long-term and short-term stability. The 6 months of postoperative stability obtained in this paper is generally considered to be the primary skeletal stability. In subsequent studies, a longer follow-up of at least 1 year postoperatively should be performed, so that the result obtained is more approximate to the final stability.

## 5. Conclusions

For the treatment of skeletal Class III malocclusion, we proposed an experimental treatment concept of surgical first approach combined with clear aligners, which was compared with the conventional orthodontic first approach by a prospective randomized controlled trial. The outcomes demonstrated that the postoperative stability of this experimental concept was not significantly different from that of the orthodontic first modality, and it was more advantageous in terms of shortening treatment time and maintaining aesthetic appearance, which is worthy of further development and application.

## Figures and Tables

**Table 1:** Baseline characteristics of the included patients.

Characteristic	Experimental Group	Control Group	*p* Value
Sex (%)			
Male	40.5	43.2	0.814
Female	59.5	56.8	
Age (mean ± SD, years)	24.62 ± 3.89	25.73 ± 4.64	0.269
Treatment time	18.05 ± 2.53	22.83 ± 3.60	0.000 *

SD, standard deviation; * *p* < 0.05, statistically significant difference.

**Table 2:** Statistic description of maxillary linear deviation and angular deviation.

Dimension		Segment	Median	IQR	*p* Value
Linear deviation (mm)	x axis	SFA	0.10	0.04–0.23	0.208
		OFA	0.08	0.03–0.15	
	y axis	SFA	0.20	0.07–0.34	0.361
		OFA	0.13	0.08–0.27	
	z axis	SFA	0.28	0.16–0.52	0.378
		OFA	0.27	0.14–1.43	
Angular deviation (°)	yaw	SFA	0.29	0.11–0.51	0.005 *
		OFA	0.16	0.06–0.23	
	roll	SFA	0.26	0.10–0.58	0.893
		OFA	0.28	0.16–0.49	
	pitch	SFA	0.71	0.34–1.30	0.2
		OFA	0.57	0.14–1.10	

IQR, interquartile range. * *p* < 0.05, statistically significant difference. SFA, surgery first approach; OFA, orthodontic first approach.

**Table 3:** Statistic description of mandibular linear deviation and angular deviation.

Dimension		Segment	Median	IQR	*p* Value
Linear deviation (mm)	x axis	SFA	0.37	0.11–0.92	0.466
		OFA	0.26	0.13–0.46	
	y axis	SFA	1.36	0.52–2.59	0.758
		OFA	1.19	0.42–2.85	
	z axis	SFA	1.08	0.41–1.59	0.402
		OFA	0.79	0.28–1.58	
Angular deviation (°)	yaw	SFA	0.37	0.23–0.67	0.075
		OFA	0.30	0.13–0.42	
	roll	SFA	0.31	0.19–0.62	0.056
		OFA	0.23	0.11–0.38	
	pitch	SFA	0.96	0.47–1.50	0.6
		OFA	0.95	0.47–1.42	

IQR, interquartile range. SFA, surgery first approach; OFA, orthodontic first approach.

## Data Availability

The data presented in this study are available on request from the corresponding author. The data are not publicly available due to privacy.
